# A Complex Inoculant of N_2_-Fixing, P- and K-Solubilizing Bacteria from a Purple Soil Improves the Growth of Kiwifruit (*Actinidia chinensis*) Plantlets

**DOI:** 10.3389/fmicb.2016.00841

**Published:** 2016-06-22

**Authors:** Hong Shen, Xinhua He, Yiqing Liu, Yi Chen, Jianming Tang, Tao Guo

**Affiliations:** ^1^Chongqing Key Laboratory of Soil Multi-scale Interfacial Process, College of Resources and Environment, Southwest UniversityChongqing, China; ^2^Collaborative Innovation Center of Special Plant Industry, Chongqing University of Arts and SciencesChongqing, China; ^3^Centre of Excellence for Soil Biology, College of Resources and Environment, Southwest UniversityChongqing, China; ^4^School of Plant Biology, University of Western AustraliaCrawley, WA, Australia; ^5^College of Forestry and Life Science, Chongqing University of Arts and SciencesChongqing, China

**Keywords:** identification, *Bacillus* spp., N_2_-fixation bacterium, K- and P-solubilizing bacteria, malondialdehyde, polyphenol oxidase

## Abstract

Limited information is available if plant growth promoting bacteria (PGPB) can promote the growth of fruit crops through improvements in soil fertility. This study aimed to evaluate the capacity of PGPB, identified by phenotypic and 16S rRNA sequencing from a vegetable purple soil in Chongqing, China, to increase soil nitrogen (N), phosphorus (P), and potassium (K) availability and growth of kiwifruit (*Actinidia chinensis*). In doing so, three out of 17 bacterial isolates with a high capacity of N_2_-fixation (*Bacillus amyloliquefaciens*, XD-N-3), P-solubilization (*B. pumilus*, XD-P-1) or K-solubilization (*B. circulans*, XD-K-2) were mixed as a complex bacterial inoculant. A pot experiment then examined its effects of this complex inoculant on soil microflora, soil N_2_-fixation, P- and K-solubility and kiwifruit growth under four treatments. These treatments were (1) no-fertilizer and no-bacterial inoculant (Control), (2) no-bacterial inoculant and a full-rate of chemical NPK fertilizer (CF), (3) the complex inoculant (CI), and (4) a half-rate CF and full CI (1/2CF+CI). Results indicated that significantly greater growth of N_2_-fixing, P- and K-solubilizing bacteria among treatments ranked from greatest to least as under 1/2CF+CI ≈ CI > CF ≈ Control. Though generally without significant treatment differences in soil total N, P, or K, significantly greater soil available N, P, or K among treatments was, respectively, patterned as under 1/2CF+CI ≈ CI > CF ≈ Control, under 1/2CF+CI > CF > CI > Control or under 1/2CF+CI > CF ≈ CI > Control, indicating an improvement of soil fertility by this complex inoculant. In regards to plant growth, significantly greater total plant biomass and total N, P, and K accumulation among treatments were ranked as 1/2CF+CI ≈ CI > CF > Control. Additionally, significantly greater leaf polyphenol oxidase activity ranked as under CF > 1/2CF+CI ≈ Control ≈ CI, while leaf malondialdehyde contents as under Control > CI ≈ CF > 1/2CF+CI. In short, the applied complex inoculant is able to improve available soil N, P, and K and kiwifruit growth. These results demonstrate the potential of using a complex bacterial inoculant for promoting soil fertility and plant growth.

## Introduction

High inputs of chemical nitrogen (N), phosphorus (P), and/or potassium (K) fertilizers have been globally used to enhance crop growth and productivity. Meanwhile, the excessive application of chemical N, P, and/or K has resulted in significantly destructive change in soil properties and environmental quality. However, studies pertaining to the relationship between the application of fertilizers and their negative effects on the environment are limited. Therefore, it is timely to explore and identify alternative strategies that can ensure competitive crop yields and environment health while maintaining agro-ecosystem sustainability (Zhang and Ju, 2002; Jiao et al., 2012). Recently, the application of microbial inoculants or plant growth-promoting (PGP) bacteria to soils has been shown to be a promising practice for sustainable production in intensive agricultural production systems around the world (Bhattacharyya and Jha, 2012; Chauhan et al., 2015; Majeed et al., 2015).

Plant growth-promoting bacteria are free-living soil bacteria that can fix or solubilize essential elements in the soil, induce plant host resistance to pathogens, and aggressively colonize the root rhizosphere. Consequently, this results in promotion of better nutrient uptake and thereby enhances plant growth and yield (Chen et al., 2006; Lugtenberg and Kamilova, 2009; Tailor and Joshi, 2014; Zhang and Kong, 2014). The effect of PGP bacteria is mostly explained by their capacity to release metabolites (Majeed et al., 2015). These metabolites are able to: (a) stimulate plant growth by inducing the production and release of plant growth regulators or phytohormones such as indole acetic acid (IAA), cytokinins, and gibberellins; (b) enhance asymbiotic N_2_-fixation; (c) solubilize inorganic phosphate and the mineralization of organic phosphate and/or other nutrients; and (d) resist, tolerate or compete with detrimental microorganisms (Lugtenberg and Kamilova, 2009). At present, the application of PGPRs in China has benefited the growth and production of rice, wheat, soybeans, corn, and potato (Chen and Zhang, 2000; Wu et al., 2011; Deng et al., 2012; Gao et al., 2012). The effectiveness of biofertilizers, such as the inoculation of soils with exogenous PGP bacteria, however, has been a hot topic of controversy because various inoculants have shown poor colonization ability, unstable genetic trait, and poor competitive advantage against indigenous soil microorganisms (Du et al., 2008). In particular, information is very limited in the effects of soil microbial inoculants in fruit plantations.

Kiwifruit (*Actinidia chinensis*) has long been recognized as ‘the king of fruits’ because of its remarkably high vitamin C and balanced mineral composition, dietary fiber, and other metabolites that are beneficial to human health. Kiwifruit is commercially grown in the Sichuan Basin of southwestern China (Li et al., 2015). This basin has an area of 2.6 × 10^5^ km^2^ and is located in eastern Sichuan (97°21′E-108°31′E, 26°03′N-34°19′N) and most of the Chongqing Municipal City (105°11′E-110°11′E, 28°10′N-32°13′N). In this region, a purple soil (Eutric Regosol, FAO Soil Taxonomic Classification) is the major agricultural soil. Some PGP bacteria, such as silicate bacteria (Huang et al., 1998; He et al., 2003) and azotobacters (Li et al., 2003; Han et al., 2011) have been isolated from this purple soil. There have been few reports, however, regarding the colonization ability of these PGP bacteria and their compatibility with soil microflora or their ability to stimulate plant growth in the purple soil.

The objectives of this study were to determine (a) if bacterial isolates from the purple soil had a competitive advantage and (b) if the use of the bacterial isolates as a complex soil inoculant could effectively solubilize soil nutrients and thus promote plant growth. In doing so, PGP bacterial isolates were first isolated from the purple soil and identified. The identified isolates were then added to purple soil for evaluating their effects on the release of soil N, P, and K, increase of plant tissue N, P, and K and plant biomass production. The expected findings of this study could increase our understanding of the use of PGP bacteria for improving soil fertility and plant growth.

## Materials and Methods

### Soil Sampling and Site Description

A total of three independent purple soil samples were collected after the harvest of soybean every week in September 2013. Fifteen randomly collected soil cores (10 mm diameter, 0–20 cm depth) were combined into one independent soil sample from each of five plots (200 m^2^), where lettuce – eggplant/red pepper – soybean – Chinese cabbage had been rotated for the past 10 years. The collected soils were kept on ice before being transported to the laboratory and stored at 4°C overnight to the next day for the isolation of PGP bacteria. The plots were located at the National Monitoring Station for Soil Fertility and Fertilizer Efficiency, close to the campus of Southwest University, Chongqing (30°26′N, 106°26′E, 266 m above the sea level), China. The area has an annual mean temperature of 18.3°C and a mean precipitation of 1,115 mm. The initial soil properties in 1991 were as follows: pH, 7.5 (water: soil = 2:1), 8.44 g organic matter kg^-1^, 0.92 g N kg^-1^, 0.54 g P kg^-1^, and 16.7 g K kg^-1^, 43.2 mg available N kg^-1^, 11.0 mg available P kg^-1^, and 65.3 mg available K kg^-1^.

### PGP Bacteria Isolation and Determinations of N_2_-fixing, P- and K-solubilizing Ability

In each bacterial isolation, a total of 10.0 g of purple soil from each plot was mixed with 90 ml sterilized tap-water and shaken at 120 rpm for 0.2 h at 28 ± 2°C (Zhang et al., 2012). This procedure was repeated five times and the obtained suspensions from each plot were mixed and then diluted with sterilized tap-water. In each 10^-4^, 10^-5^, 10^-6^, or 10^-7^ dilution, 100 μl suspensions were incubated in each plate containing N_2_-fixing Ashby, P-solubilizing PKO or K-releasing agar medium for 3–5 days at 28°C (Zhang et al., 2012). Bacterial colonies were then picked up, according to growth speed and transparent zones around, for further purification. All purified isolates were cryopreserved at -80°C in LB containing 40% glycerol. Four replicated bacterial isolations from each plot were performed to evaluate the bacterial population (CFU g^-1^ soil, Colony Forming Units).

Observation of the presence of N_2_-fixing, P- and K-solubilizing bacteria was accorded to Perez et al. (2007), Akhter et al. (2012), and Yi et al. (2012), respectively. Briefly, three replicates of 2 ml purified N_2_-fixing, P- or K-solubilizing bacterial isolates (10^8^ CFU/ml) were, respectively, added to 100 ml of corresponding medium and incubated at 28 ± 2°C and 120 rpm for 7 days. Then, 10 ml supernatant from each of these incubated cultures were then collected after centrifugation at 5,000 rpm for 10 min. Determination of N, P, and K content in the collected supernatant was accorded to the micro-kjeldahl method (Hendershot, 1985), Mo-Sb colorimetric method (Perez et al., 2007), and flame spectrophotometer method (Yi et al., 2012), respectively. The above-mentioned procedure was repeated three times or batches. A total of 17 bacterial isolates were then initially screened according to their growth curve, pH and temperature tolerance, and N, P, and K content (data not shown). These 17 bacterial isolates included six for N_2_-fixing (named as XD-N-1 to XD-N-6), six for P-solubilizing (XD-P-1 to XD-P-6), and five for K-solubilizing (XD-K-1 to XD-K-5) ones.

### Phenotypic Identification and 16s rRNA Sequencing of the Obtained Isolates

The determination of phenotypically physiological and biochemical characteristics of these 17 bacterial isolates was accorded to (Samina et al., 2010). These characteristics included cell shape and size, colony pigmentation, Gram staining, spore formation, motility and UV-fluorescence (data not shown), and gelatin liquefaction, glucose produced acid and gas, indole production, methyl red test, nitrate reduction, presence of catalase and oxidase, phenylalaninase, starch hydrolysis, use of citrate, Voges–Proskauer test and xylose produced acid (see data in **Table 1** for three selected isolates).

**Table 1:** Physiological and biochemical characteristics of the three isolates obtained from a purple soil.

Characteristics	XD-N-3	XD-P-1	XD-K-2
Catalase	+	+	+
Gelatin liquefaction	+	+	+
Glucose produced acid	+	+	+
Glucose produced gas	-	-	-
Indole production	-	-	-
Methyl red test	+	-	+
Nitrate reduction	+	-	+
Oxidase	-	-	+
Phenylalaninase	-	-	-
Starch hydrolysis	+	-	+
Use of citrate	-	+	+
Voges-Proskauer test	+	+	-
Xylose produced acid	+	+	+

*“+” and “-” indicate positive or negative reaction, respectively. Isolate code: XD-K-2, K-solubilizing strain; XD-N-3, N_2_-fixing strain 3; XD-P-1, P-solubilizing strain. See **Table 2** for abbreviation details.*

After the phenotypic determination, the genomic DNA of these 17 isolates was extracted using a TAKARA Mini BEST Bacteria Genomic DNA Extraction Kit Ver.3.0 (TAKARA BIO INC.). DNA quality was assessed by OD 260/280 and 260/230 ratio (Beckman Coulter DU 800) and amplified using the universal bacterial 16S rRNA forward primer 27F (5′-AGAGTTTGATCATGGCTCAG-3′) and reverse primer 1492R (5′-TACGGTTACCTTGTTACGACTF-3′). With a *Premix Taq^TM^* Kit (*TAKARA Taq^TM^* Version 2.0 plus dye, Takara Bio, Otsu, Japan), the PCR was performed using an ABI PCR Instrument (GenenAmp^®^ 9700). The PCR mixture contained 25.0 μl *Premix Taq^TM^* (1.25U DNA polymerase, 4 mM Mg^2+^, 0.4 mM dNTP mixture), 1.0 μl 20 μM forward primer, 1.0 μl 20 μM reverse primer, 1.0 μl template DNA (about 100 ng), and 22.0 μl nuclease-free water to a final volume of 50 μl. After the initial denaturation for 5 min at 95°C, the PCR running was 30 cycles of denaturation for 60 s at 95°C, annealing for 60 s at 55°C, and elongation for 2 min at 72°C, except for 10 min in the final elongation. The PCR products were recovered using an AxyPrepDNA Gel Extraction Kit (Axygen Bio, USA) following the manufacturer’s instructions. The amplified partial 16S rRNA gene was a single band (1,300–1,500 bp) by electrophoresis through a 2.0% (w/v) agarose gel and 0.5 mg L^-1^ ethidium bromide. The PCR products were commercially quantified by the Invitrogen Trading Co., Ltd. (ShangHai, China) using the QuantiFluor^TM^-ST blue fluorescence quantitative system (Promega, China).

The obtained gene sequences were compared with other sequences and the accession numbers (see below in the Result section) were deposited in the GenBank database using the NCBI BLAST^1^. Multiple sequence alignments were performed by CLUSTAL W (MEGA Version 6.06). The phylogenetic tree topology based on re-samplings of 1000 times of the neighbor joining data set was evaluated by the boot strap analysis (MEGA Version 6.06).

### Plant Growth Experiment

The selected three isolates of XD-N-3, XD-P-1, and XD-K-2 from the initial 17 screened isolates were used as an inoculant in the plant growth experiment. In doing so, the suspension of XD-N-3, XD-P-1, and XD-K-2, after being separately cultured in LB medium, were mixed together with a ratio of 1:1:1 of CFU into 1.0 kg autoclaved peat (<2 mm) as a complex inoculant (Xi et al., 2009). The total number of effective bacteria was about 3 × 10^8^ cfu.g^-1^ in this complex inoculant. The peat (pH 6.7) contained 437 g organic matter kg^-1^, 10.2 g N kg^-1^, 2.23 g P kg^-1^, and 6.42 g K kg^-1^.

Four treatments were administered to air-dried and sieved (2 cm) purple soil (**Table 2**): (1) no-fertilizer and no-bacteria inoculant (Control), (2) no-bacteria inoculant and a full-rate of chemical NPK fertilization (CF, N:P:K = 2.6:1:1) as urea (321 mg N kg^-1^), Ca (H_2_PO_4_)_2_⋅H_2_O (123.5 mg P kg^-1^), and KCl (123.5 mg K kg^-1^), (3) a mixture of the three bacteria as a complex inoculant (CI), and (4) a half-rate of chemical NPK and a full dose of the complex bacterial inoculant (1/2CF+CI). The soil was placed in pots (10 cm × 10 cm, each contained 1.5 kg⋅soil) and each treatment was represented by four replicates using a fully randomized design. Two uniform, virus-free, tissue-cultured kiwifruit (*A. chinensis* cv. ‘Hongyang’) plantlets were transplanted into each pot, grown in a greenhouse for 50 days at the Chongqing University of Arts and Science, and then for an additional 40 days in a greenhouse at the College of Resources and Environment, Southwest University, Chongqing, China. Soil moisture was regularly adjusted to 55% water holding capacity with deionized water during the whole period of plant growth. The study was conducted during the spring of 2014.

**Table 2:** The nutrient composition (mg/kg) of soil treatments utilized in the present study to determine the effect of a complex bacterial inoculants on soil fertility and plant growth.

Treatment	Urea	Calcium superphosphate	Potash	PGP bacterial inoculate
Control	0	0	0	0
CF	321	123.5	119	0
CI	0	0	0	30
1/2CF+CI	160.5	61.75	59.5	30

*Control, no-fertilizer and no-inoculant control; CF, no-inoculant and full dose of chemical NPK fertilization; CI, full bacterial complex inoculant; 1/2CF+CI, half-dose of chemical NPK fertilization and full bacterial complex inoculant.*

### Analyses of Soil and Plant Samples from the Plant Growth Experiment

Soil samples from each pot were randomly collected around the fine roots of the two kiwifruit plantlets and kept on ice during transport to the laboratory where they were stored overnight at 4°C prior to conducting CFU of bacterial counting analyses. Soil samples for the determination of chemical properties were sieved (1 mm) after being air-dried at room temperature for 7 days and then stored at 4°C prior to analysis. Determinations of soil organic matter (SOM; digestion method), total N, P, and K (micro-Kjeldahl digestion), available N (alkaline diffusion method), available P (0.5 M NaHCO_3_-extractable P, pH 8.5), available K (1.0 M NH_4_OAc)-extractable K) and soil pH (soil:water = 1:1, v/v) were accorded to Page et al. (1982).

At harvest, plant height, ground diameter, and total leaf area were measured. Leaves were collected as described by Lamb and Rubery (1976). Oven-dried (48 h at 70°C) root and shoot samples were weighed and milled with a high-speed multi-function micro-pulverizer (Whirl Type Model Y-60, Hebei, China) prior to analysis. Plant samples were digested by a mixture of 96% H_2_SO_4_ and 30% H_2_O_2_. With a Shimadzu Model UV-120-02 spectrophotometer, the digested solution was analyzed for plant N by the Kjeldahl method, P by the molybdate blue colorimetric analysis, and K by the flame photometry (Nanjing Agricultural University, 1994). Leaf polyphenol oxidase (PPO) activity and malondialdehyde (MDA) content were assayed according to Moerschbacher et al. (1988) and Hodges et al. (1999), respectively.

### Data Analyses

The pot experiment was only performed once, i.e., without a biological replicate, but with four replicates. Data were hence expressed as means ± SD (*n* = 4) and subjected to ANOVA, and significant differences between treatment means were then compared by the Tukey High Significant Difference (Tukey HSD) Test at *P* ≤ 5% using SPSS (Version 19.0, New York, USA).

## Results and Discussion

### Identification and Characterization of Bacterial Isolates

**FIGURE 1:**
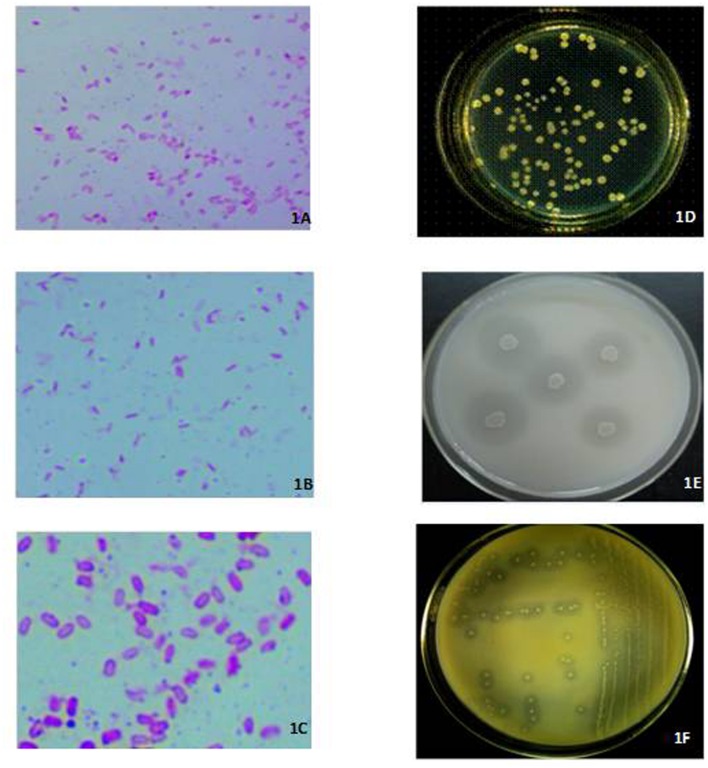
**Left: Photomicrographs (1600×) of cells and spores of XD-N-3 (A), XD-P-1 (B), and XD-K-2 (C) grown on LB medium at 28°C for 3 days.** Right: *in vitro* colonial morphology of XD-N-3 **(D)** grown on the Ashby medium at 28 ± 2°C after 3 days, and XD-P-1 **(E)**, XD-K-2 **(F)** respectively, grown on the Pikovskaya’s and K-amended selective medium at 28 ± 2°C after 5 days. The formation of halo zone around the colonies of **(E)** and **(F)** showed the solubilization of the inorganic phosphate and potassium.

Three isolates, respectively, identified by their N_2_-fixing, and P- and K-solubilizing capacity as XD-N-3, XD-P-1, and XD-K-2 through phenotypic identification and 16s rRNA sequencing, were combined to form a complex inoculant that was used in this study. The N_2_-fixing, and P- and K-solubilizing capacity of these three selected bacterial isolates was 91.5 ± 2.69 mg N L^-1^ for the N_2_-fixing XD-N-3, 129 ± 11.89 mg P L^-1^ for the P-solubilizing XD-P-1, and 23.2 ± 0.79 mg K L^-1^ for the K-solubilizing XD-K-2. The biochemical characteristics of these three isolates are presented in **Table 1**. All three isolates were fast growing, Gram-positive, and appeared as small, oval rods with a mid-spore, and a thickening capsule (see **Figures 1A–C**). In general, colonies of XD-N-3 were white, semitransparent, regular shaped with a raised elevation and a smooth surface. The average colony diameter was 2–3 mm in 2 days and 6–8 mm in 5 days on Ashby medium (**Figure 1D**). Colonies of XD-P-1 formed an obvious P-solubilizing circle and their average diameter was 2–3 mm in 2 days and 5–6 mm in 5 days on Pikovskaya’s medium. The colonies had regular margins, and were moist, transparent, and hard to pick out (**Figure 1E**). Colonies of XD-K-2 formed an obvious K-solubilizing circle and had an average diameter of -2 mm in 2 days and 5–7 mm in 5 days on K-amended selective medium. The colonies were moist, milky white, semitransparent, regular shaped with raised elevation and a smooth surface (**Figure 1F**).

**FIGURE 2:**
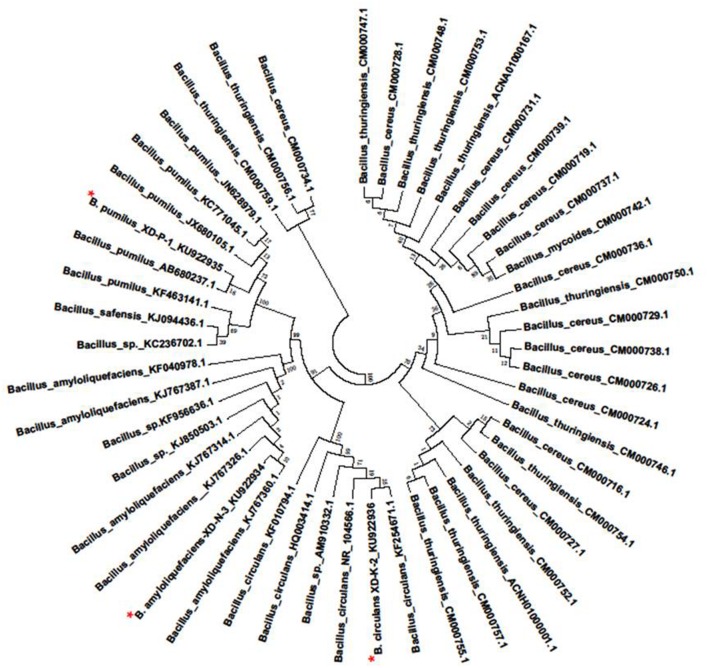
**Phylogenetic tree was prepared by Neighbor-joining method of the XD-N-3, XD-P-1, and XD-K-2 which was followed by an asterisk, respectively.** Numbers above or below the nodes indicate bootstrap values generated after 1000 replications. Bacteria species and sequences from GeneBank were shown with their accession numbers in the figure. The evolutionary distance was computed using the Maximum Likelihood method and was in the units of the number of base substitutions per site.

The sequences analysis of 1.5 kb fragment of 16S rRNA genes of the three selected bacterial isolates (XD-N-3, XD-P-1, and XD-K-2) were aligned with other sequences in the GenBank database. The phylogenetic tree of the three bacterial isolates constructed by using their 16s rRNA (**Figure 2**) showed that they were members of genus *Bacillus*. With a similarity of 99, 99, and 98% to respective *Bacillus amyloliquefaciens*, *B. pumilus*, and *B. circulans*, the 16s rRNA gene nucleotide sequence of these three isolates were designated as *B. amyloliquefaciens* XD-N-3, *B. pumilus* XD-P-1, and *B. circulans* XD-K-2, respectively. These isolates were submitted to GeneBank under the accession number KU922934, KU922935, and KU922936, respectively.

*Bacillus* is one of the most commonly studied genera in plant and soil sciences (Hallmann and Berg, 2006). The isolates belonging to *Bacillus* identified in the present study was similar to these identified in root adhering soil of various host plants including peas, clovers (Laguerre et al., 1994). The colonization of the rhizosphere by *Bacillus* sp. during the active plant growth has been reported in maize in France (Lambert et al., 1987) and in Canada (Lalande et al., 1989), in a number of crops inclduing buckwheat, finger millet, frenchbean, and maize (Pal, 1998) and tomato (Mehta et al., 2015) in India, and in wheat in Pakistan (Majeed et al., 2015).

### Effects of the Complex Inoculant on the Population of Soil Bacteria

According to above-mentioned methods, three isolates, i.e., XD-N-3, XD-P-1, and XD-K-2, were selected as potential PGP bacteria and combined to produce a complex inoculant. After 90 days of kiwifruit plantlets grown in greenhouse, the level of bacterial growth in the four soil treatments was ranked from highest to lowest as follows: 1/2CF+CI > CI > CF ≈ Control for total CFUs, and 1/2CF+CI ≈ CI > CF ≈ Control for N_2_-fixing, P-solubilizing -and K-solubilizing bacteria (**Table 3**). These results indicate that there were no significant differences between the Control and CF treatment in regards to total CFUs or the level N_2_-fixing, P-, and K-solubilizing bacteria (**Table 3**). Compared to the Control, however, the CFUs of total culturable bacteria, N_2_-fixing, P- and K-solubilizing bacteria were strongly enhanced in the CI treatment by 915, 5046, 969, and 729%, respectively. Although the CFUs of culturable bacteria were significantly higher in the 1/2CF+CI treatment than in the CI treatment, there were no significant differences in the CFUs of either N_2_-fixing, P- or K-solubilizing bacteria. Additionally, the level of the N_2_-fixing, and P/K-solubilizing bacteria were ten-fold greater in the 1/2CF+CI treatment than in the Control and CF treatments. Since the populations of N_2_-fixing bacteria, as well as P- and K-solubilizing bacteria were significantly higher in the 1/2CF+CI treatment, these results evidenced that the three isolates, namely *B. amyloliquefaciens* XD-N-3, *B. pumilus* XD-P-1 and *B. circulans* XD-K-2, had ecological adaptation of the tested purple soil.

**Table 3:** Effect of the complex inoculant on the populations of culturable bacteria in a purple soil.

Treatment	Total Bacteria (×10^6^cfu •g^-1^ soil)	N_2_-fixing bacteria	P-solubilizing bacteria	K-solubilizing bacteria
		
			(×10^3^ cfu •g^-1^ soil)	
Control	3.98 ± 2.28ˆc	0.55 ± 0.31ˆb	3.48 ± 2.28ˆb	7.95 ± 4.56ˆb
CF	11.63 ± 2.90ˆc	1.93 ± 0.84ˆb	6.76 ± 3.02ˆb	13.53 ± 6.03ˆb
CI	40.42 ± 4.95ˆb	28.33 ± 11.62ˆa	37.24 ± 8.92ˆa	65.93 ± 6.01ˆa
1/2CF+CI	64.52 ± 16.05ˆa	30.52 ± 10.77ˆa	34.81 ± 10.00ˆa	55.90 ± 1.57ˆa

*Data (means ± SD, n = 4) followed by different letters in the same column (a,b) between treatments indicate significant differences at P ≤ 0.05. See **Table 2** for abbreviation details.*

### Effects of the Complex Inoculant on the Availability of Soil Nutrients

**FIGURE 3:**
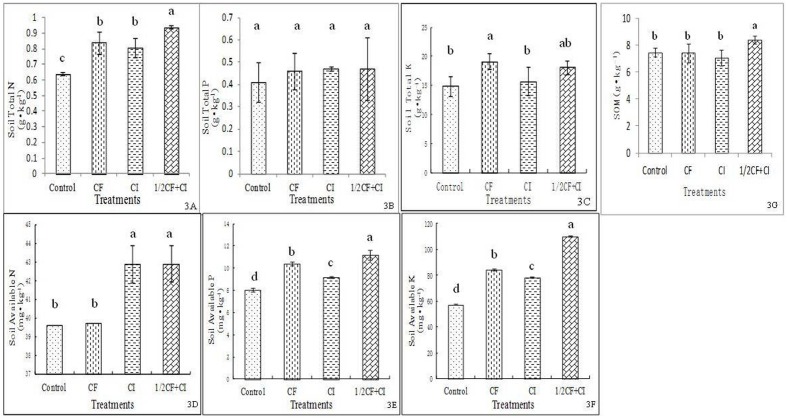
**Effect of the complex inoculant on soil total N (A), total P (B) and total K (C), and soil available N (D), available P (E), available K (F) and soil organic matter (G) after 90 days of kiwifruit plantlets grown under greenhouse conditions.** Data (means ± SD, *n* = 4) followed by different letters in the same column (a, b) between treatments indicate significant differences at *P* ≤ 0.05. Control, no-fertilizer and no-inoculant control; CF, no-inoculant and full dose of chemical NPK fertilization; CI, full bacterial complex inoculant; 1/2CF+CI, half-dose of chemical NPK fertilization and full bacterial complex inoculant.

The level of soil available N (**Figure 3D**), P (**Figure 3E**), and K (**Figure 3F**) differed significantly between the Control and the CI treatments, indicating that an improvement in soil fertility was achieved through the inoculation of this complex inoculant. The improved level of availability of soil N, P, and K was consistent with results reported in soil grown alfalfa (Han et al., 2011) and vegetable crops of tomato and spinach rotation system (Song et al., 2015). In contrast to our complex inoculant (*B. amyloliquefaciens* XD-N-3, *B. pumilus* XD-P-1, and *B. circulans* XD-K-2), a mixed inoculant of azotobacters, P- and K-solubilizing bacteria was applied by Han et al. (2011), and a mixed inoculant of two strains of *B. subtilis* and two strains of *B. mucilaginosus* was employed by Song et al. (2015).

Significantly differences in SOM were observed between treatments ranked from highest to lowest as follows: 1/2CF+CI (8.38 g⋅kg^-1^) > CI ≈ CF ≈ Control (**Figure 3G**). Significant differences in total soil nutrient levels were observed between treatments ranked from highest to lowest as follows: 1/2CF+CI (0.94 g N kg^-1^) ≈ CI ≈ CF > Control for total N (**Figure 3A**), 1/2CF+CI (0.47 g P kg^-1^) ≈ CI ≈ CF ≈ Control for total P (**Figure 3B**), and 1/2CF+CI (18.14 g K kg^-1^) ≤ CF > CI ≈ Control for total K (**Figure 3C**). A pattern of increasing soil available nutrients was observed between the treatments ranked from highest to lowest as follows: 1/2CF+CI (42.93 mg N kg^-1^) ≈ CI > CF ≈ Control for available N, 1/2CF+CI (11.18 mg P kg^-1^) > CF > CI > Control for available P, and 1/2CF+CI (110.42 mg K kg^-1^) > CF ≈ CI > Control for available K. Collectively, these results indicated that the complex inoculant had the capacity to increase soil available N, P, and K (CI vs. Control). In addition, the combination of a half-rate of chemical fertilizers with the addition of the complex inoculants containing the three PGP bacterial isolates may have the potential to replace the amount of chemical fertilizer needed to obtain optimum levels of essential nutrients. Importantly, no significant increases in total soil N, P, and K concentrations were observed between the four treatments. Caution should be taken, however, in regards to this claim since the effect of just a 1/2 CF treatment was not examined in the present study. Rong et al. (2014) reported that a biofertilizer consisting of five P-solubilizing isolates and one rhizobium isolate could replace 80% of the chemical fertilizer and produce a similar yield in pea. These results suggested that the inoculated PGP bacteria had contributed to the solubilization of P and/or K either from the soil itself or from the applied chemical P and K fertilizer. The results in N_2_-fixation and P-solubilization in the present study are in good accordance with the increase of plant tissue N in maize and soil P availability obtained by three bacterial isolates that showed N_2_-fixation and P-solubilizing ability (Zahid et al., 2015). Among these three isolates, two showed 99% similarity with *B. subtilis* and another 99% similarity with *B. megaterium*. The maize was gown under greenhouse conditions along with or without *Bacillus* inoculation, and 1/2NP and full NP fertilization (60 and 45 mg kg^-1^ soil for full N and P rate).

### Effects of the Complex Inoculant on Plant Growth

**FIGURE 4:**
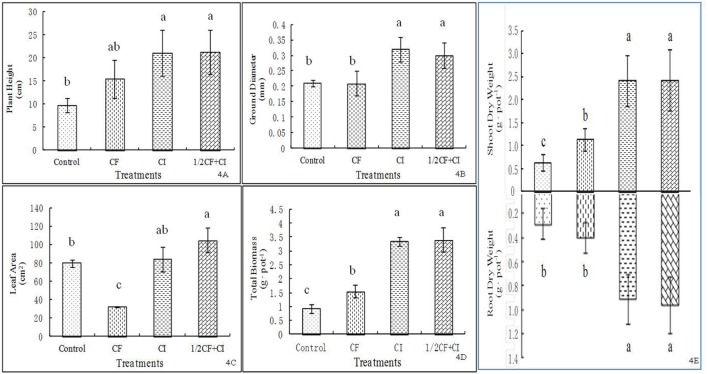
**Effect of the complex inoculant on plant height (A), plant ground diameter (B), leaf area (C), total biomass (D) and shoot and root dry weigh (E) of kiwifruit plantlets after 90 days grown under greenhouse conditions.** Data (means ± SD, *n* = 4) followed by different letters in the same column (a, b) between treatments indicate significant differences at *P* ≤ 0.05. Control, no-fertilizer and no-inoculant control; CF, no-inoculant and full dose of chemical NPK fertilization; CI, full bacterial complex inoculant; 1/2CF+CI, half-dose of chemical NPK fertilization and full bacterial complex inoculant.

A pattern of an increasing positive impact was observed among treatments ranked from greatest to least as follows: 1/2CF+CI (21.3 cm) ≈ CI > CF > Control for plant height (**Figure 4A**), 1/2CF+CI (0.3 mm) ≈ CI > CF ≈ Control for stem diameter at the ground surface (**Figure 4B**), 1/2CF+CI (104.6 cm^2^) > CI > Control > CF for leaf area (**Figure 4C**), 1/2CF+CI (2.43 g⋅pot^-1^ and 3.40 g⋅pot^-1^) ≈ CI > CF > Control for both shoot DW and total plant total biomass, respectively (**Figure 4D**), and 1/2CF+CI (0.97 g⋅pot^-1^) ≈ CI > CF ≈ Control for root biomass (**Figure 4E**). In comparison to the Control, plant height, and shoot and root dry weight were significantly increased by 59.8, 83.3, and 33.3% under CF, respectively; while leaf area was significantly decreased by 59.2%.

One of the multiple pathways that PGPRs promote plant growth is to manipulate root growth (Biswas et al., 2000; Lucy et al., 2004). The application of PGP bacteria (CI) resulted in a fairly strong growth-promoting effect on kiwifruit plantlets. For example, a 200 and 125% higher root biomass production was observed under CI, in compared to the Control and CF treatments, respectively. Also compared to the Control, shoot dry weight and plant height both increased significantly by 300 and 118% under CI and by 50 and 50% under CF, respectively.

Interestingly, both shoot and root dry biomass was significantly increased by 118 or 150% under 1/2CF+CI and CF, respectively, compared to the Control (**Figure 4E**). A similar trend was observed between the CI and CF treatments. These results are in accordance with results of plant biomass production obtained in maize (Zahid et al., 2015), Chinese kale (Piromyou et al., 2013) and almost all major vegetables and fruits (Berg, 2009). For instance, compared to the un-inoculated plant, biomass production of maize was increased by seven *Bacillus* isolates that showed ability to promote N_2_-fixation, P-solubilizing or indole-3-aceticacid production, no matter whether the maize was gown under 1/2NP or full NP fertilization (60 and 45 mg kg^-1^ for full N and P rate) in a greenhouse (Zahid et al., 2015). Similarly, under the same fertilization rate of 1,000 kg compost containing 10 mg N, 29 g P, and 10 mg K kg^-1^, and 130 kg N, 80 kg P, and 60 kg K ha^-1^ to both pot and field experiments, no effects of the sole compost on plant biomass, but the dual application of compost with *Bacillus* sp. SUT1 did enhance the biomass production of Chinese kale, particularly in the field experiment, indicating a direct growth promotion by *Bacillus* sp. SUT1 (Piromyou et al., 2013). In addition, a number of *Bacillus* isolates, e.g., *B. lichenformis*, *B. megaterium*, *B. subtilis* FZB24, *B. subtilis* GB03, *B. pumilus* GB34, and *B. subtilis* QST716, had been used to not only to promote plant biomass production (almost all major vegetables and fruits), but also to control a variety of bacterial and fungal pathogens (*Botrytis*, *Fusarium*, *Phytophthora*, *Phomopsis*, *Pythium*, and *Rhizoctonia*, etc.; see a review by Berg, 2009). Collectively these studies suggested that the inoculation of soils with PGP bacteria could enhance fertilizer use efficiency (Adesemoye and Kloepper, 2009) by decreasing the level of chemical fertilizers that were required to obtain greater growth and yield (Adesemoye et al., 2009; Rong et al., 2014; Zahid et al., 2015).

**FIGURE 5:**
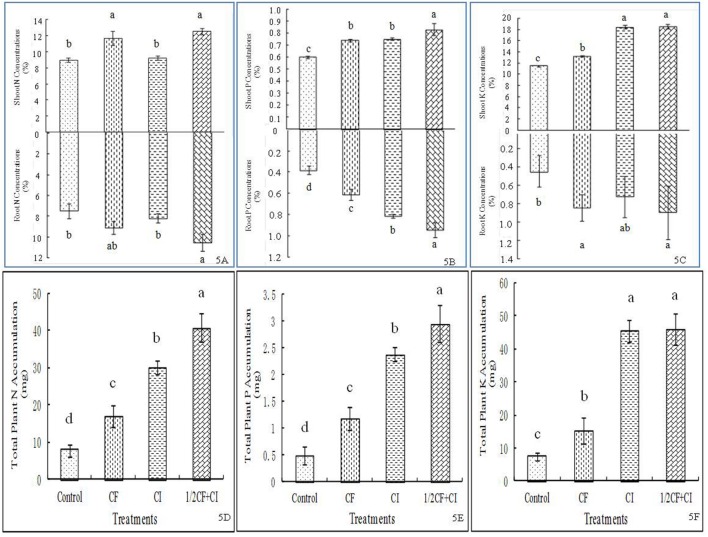
**Effect of the complex inoculant on concentrations of plant N (A), P (B), K (C) and accumulations of total N (D), P (E), and K (F) of kiwifruit plantlets after 90 days grown under greenhouse conditions.** Data (means ± SD, *n* = 4) followed by different letters in the same column (a, b) between treatments indicate significant differences at *P* ≤ 0.05. Control, no-fertilizer and no-inoculant control; CF, no-inoculant and full dose of chemical NPK fertilization; CI, full bacterial complex inoculant; 1/2CF+CI, half-dose of chemical NPK fertilization and full bacterial complex inoculant.

The highest levels of N (**Figure 5A**), P (**Figure 5B**), and K (**Figure 5C**) in both shoots and roots were always observed under 1/2CF+CI; with the exceptions for shoot and root N under CF, shoot P under CF and CI, and shoot K under CI. Compared to the CF treatment, N, P, and K concentrations under 1/2CF+CI were increased by 6.8, 12.0, and 40.9% in shoots, or by 16.1, 32.2, and 29.1%, in roots, respectively. Meanwhile, plant total N (**Figure 5D**), P (**Figure 5E**), and K (**Figure 5F**) accumulation in the treatments generally ranked from highest to lowest as follows: 1/2CF+CI (40.79 mg N, 2.95 mg P and 46.01 mg K) ≈ CI > CF > Control, though total plant N accumulation was similar between the CF and CI treatments. These results in kiwifruit are generally consistent with these in pepper and cucumber (Han and Lee, 2006), red pepper (Islam et al., 2013), and sugar beet (Shi et al., 2011). In contrast to our triple inoculation with *B. amyloliquefaciens* XD-N-3, *B. pumilus* XD-P-1, and *B. circulans* XD-K-2, the inoculation with both P- and K-solubilizing *B. megaterium* var. *phosphaticum* and *B. mucilaginosus* resulted in significantly greater soil P and K availability (Han and Lee, 2006). Meanwhile, inoculation with the N_2_-fixing bacteria *Pseudomonas* sp. RFNB3 (Islam et al., 2013) and *Acinetobacter johnsonii* strain 3–1 (Shi et al., 2011) had resulted in significantly greater uptake of N, P, and K, shoot and root biomass production. Collectively, these studies from this study and other studies indicate that PGP bacteria, used as biofertilizers, have the potential to reduce the application rate of chemical fertilizers. Caution should be taken to this claim, however, since the sole application of the 1/2 CF treatment was not examined in the present study.

### Effects of the Complex Inoculant on Leaf PPO Activity and MDA Content

**FIGURE 6:**
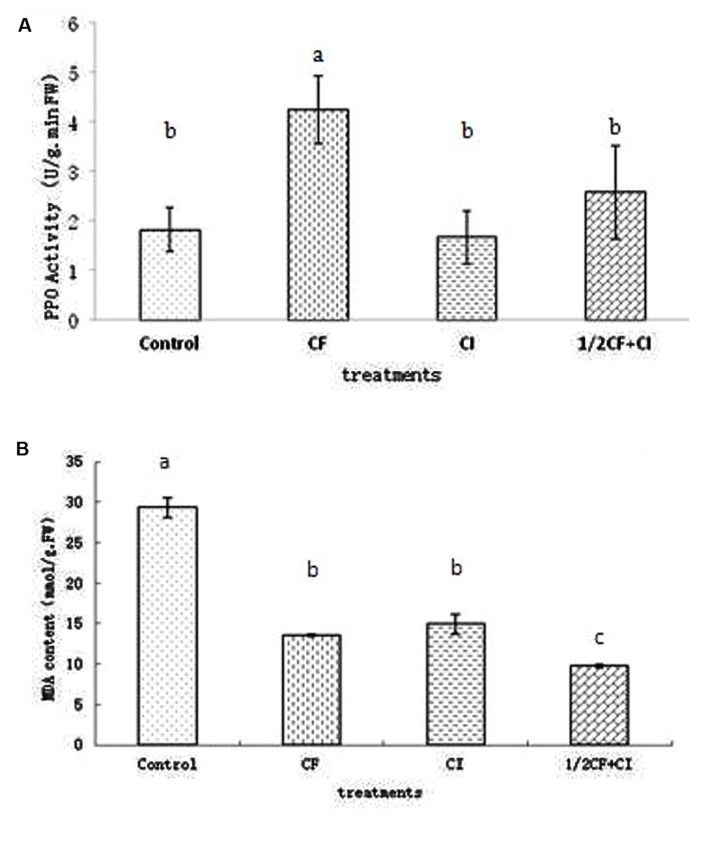
**Activity of leaf polyphenol oxidase (PPO; A) and content of Malondialdehyde (MDA; B) in 90-days-old kiwifruit seedlings.** Data (means ± SD, *n* = 4) followed by different letters (a, b) between treatments indicate significant differences at *P* ≤ 0.05.

Leaf PPO activity between treatments ranked from significantly highest to lowest as follows: CF (4.24 U/g⋅min FW) > 1/2CF+CI (2.58 U/g⋅min FW) > Control (1.81 U/g⋅min FW) ≈ CI (1.67 U/g⋅min FW; **Figure 6A**). PPO is a plant respiratory chain terminal oxidase, which catalyzes the oxidation of phenolic compounds to highly toxic quinones that play an important role in plant disease resistance (Richter et al., 2012). Thus the relatively greater leaf PPO activity under 1/2CF+CI might indicate the potential to apply this complex inoculant to enhance plant disease resistance. However, leaf PPO activity is also induced by plant pathogens such as the early blight disease pathogen in tomato (Narendra et al., 2015), the Panama Wilt Resistance pathogen in banana (Kavino et al., 2014), and the *Fusarium*-wilt pathogen in eggplant and tomato (Altinok et al., 2013; Mei et al., 2014).

Leaf MDA between treatments ranked from significantly highest to lowest as follows: Control (29.33 mmol/g⋅FW) > CI (14.94 mmol/g⋅FW) ≈ under CF (13.61 mmol/g⋅FW) > under 1/2CF+CI (9.79 mmol/g⋅FW; **Figure 6B**). In general, MDA content is negatively correlated with plant stress resistance and increases in response to stress conditions such as soil salinization (Shukla et al., 2012; Xun et al., 2015), and drought (Wang et al., 2012). Thus, the comparatively lower leaf MDA content in kiwifruit plantlets under 1/2CF+CI and CI might suggest that this complex bacterial inoculant could provide a protective effect to plants under stress conditions.

## Conclusion

Three selected PGP bacterial isolates, *B. amyloliquefaciens* XD-N-3, *B. pumilus* XD-P-1, and *B. circulans* XD-K-2, which were isolated from a purple soil, exhibited a strong capacity to fix N_2_, and solubilize P and K, respectively. Results from a pot experiment with kiwifruit plantlets demonstrated the positive effects of the complex inoculant (a mixed combination of these three *Bacillus* isolates). Specifically, increased available soil N, P, and K in purple soil were observed, in addition to a promotion of leaf N, P, and K levels. Consequently, these effects resulted in an increased plant biomass, leaf PPO activity and MDA. Results of the present study indicated that this complex inoculant might have potential to be used as a bio-fertilizer for improving soil fertility and plant growth. Future studies should focus on (a) the development of a formulation for the complex inoculant that optimizes the ratio of inoculum and the carrier (peat) matrix; (b) a complete biosafety analysis of the use of PGP bacteria agents in field applications.

## Author Contributions

HS wrote the manuscript. XH School of Plant Biology, University of Western Australia, Crawley 6009, Australia, re-written this manuscript. YL director of Collaborative Innovation Center of Special Plant Industry, this project sponsor. YC and JT carried out experiments. TG Lab manager of Chongqing Key Laboratory of Soil Multi-scale Interfacial Process, designed experiments.

## Conflict of Interest Statement

The authors declare that the research was conducted in the absence of any commercial or financial relationships that could be construed as a potential conflict of interest.

## Footnotes

1 http://www.ncbi.n1m.nih.gov/blast/Blast.cgi
